# Cytological Perspective in a Case of Doege-Potter Syndrome With Hypoinsulinemic Hypoglycemia

**DOI:** 10.7759/cureus.56041

**Published:** 2024-03-12

**Authors:** Ingitha Pulikkal, Mahadev Meena, Garima Goel, Abhishek Goyal, Tanya Sharma

**Affiliations:** 1 Pathology and Laboratory Medicine, All India Institute of Medical Sciences, Bhopal, Bhopal, IND; 2 General Medicine, All India Institute of Medical Sciences, Bhopal, Bhopal, IND; 3 Pulmonary Medicine and Tuberculosis, All India Institute of Medical Sciences, Bhopal, Bhopal, IND; 4 Respiratory Medicine, Graphic Era Institute of Medical Sciences, Dehradun, Dehradun, IND

**Keywords:** paraneoplastic syndromes, hypoglycemia, cytomorphology, solitary fibrous tumor, mesenchymal neoplasm

## Abstract

Solitary fibrous tumor (SFT) of the lung is a rare mesenchymal neoplasm of uncertain histogenesis, unknown molecular features, and unpredictable clinical behavior, characterized by NAB2-STAT6 fusion. Hypoglycemia accompanying SFT (Doege-Potter syndrome) is an uncommon presentation. We present the cytomorphological features on biopsy imprint smears of a histopathologically confirmed case of SFT of the lung with an uncommon presentation. A 76-year-old non-smoker, non-alcoholic, and non-diabetic man presented with complaints of intermittent episodes of confusion with syncopal attacks (>10 episodes) for six months. The patient had no respiratory complaints and no history of weight loss. Laboratory investigations revealed fasting blood sugar of 38 mg/dl with low serum insulin and C-peptide levels. Physical examination revealed reduced air entry on the left side of the chest. Chest X-ray showed left-sided homogenous opacity. High-resolution computed tomography (HRCT) of the chest showed a large left-sided lung mass. A biopsy was performed. Biopsy imprint smears were cellular and showed tumor cells arranged in clusters and fragments with traversing capillaries displaying monomorphic pump to oval nuclei, fine granular evenly dispersed chromatin, regular nuclear membrane, inconspicuous nucleoli, and a moderate amount of wispy cytoplasm. Foci of intercellular hyaline stromal material were noted. A cytodiagnosis of low-grade mesenchymal neoplasm was made. Histopathology revealed a cellular tumor comprising tightly packed round to fusiform cells arranged around blood vessels with intervening thick collagen, positive for CD99, vimentin, BCL2, CD34, and STAT6 and negative for EMA, CK AE1/AE3, S100, TLE1, and SMA. Familiarity with cytomorphology plays a pivotal role in clinching an early diagnosis of this rare neoplasm of the lung, particularly in the setting of presentation with hypoglycemia.

## Introduction

Solitary fibrous tumor (SFT) associated with non-islet cell tumor hypoglycemia (NICTH) is referred to as Doege-Potter syndrome [1,2]. It is a rare paraneoplastic syndrome presenting as hypoinsulinemic hypoglycemia from the ectopic production of incompletely processed form or prohormone of insulin-like growth factor 2 (IGF-2) by the SFT of the lung [3,4]. It is seen in about 4% of SFT cases. SFT of the lung is a rare mesenchymal neoplasm of uncertain histogenesis, unknown molecular features, and unpredictable clinical behavior. It is characterized by NAB2-STAT6 fusion [5]. We present the cytomorphological features on imprint smears of a histopathologically confirmed case of SFT of the lung with an uncommon presentation.

This article was previously presented as a poster at CYTOCON 2021, Delhi Chapter, held on November 19-21, 2021.

## Case presentation

**Figure 1:**
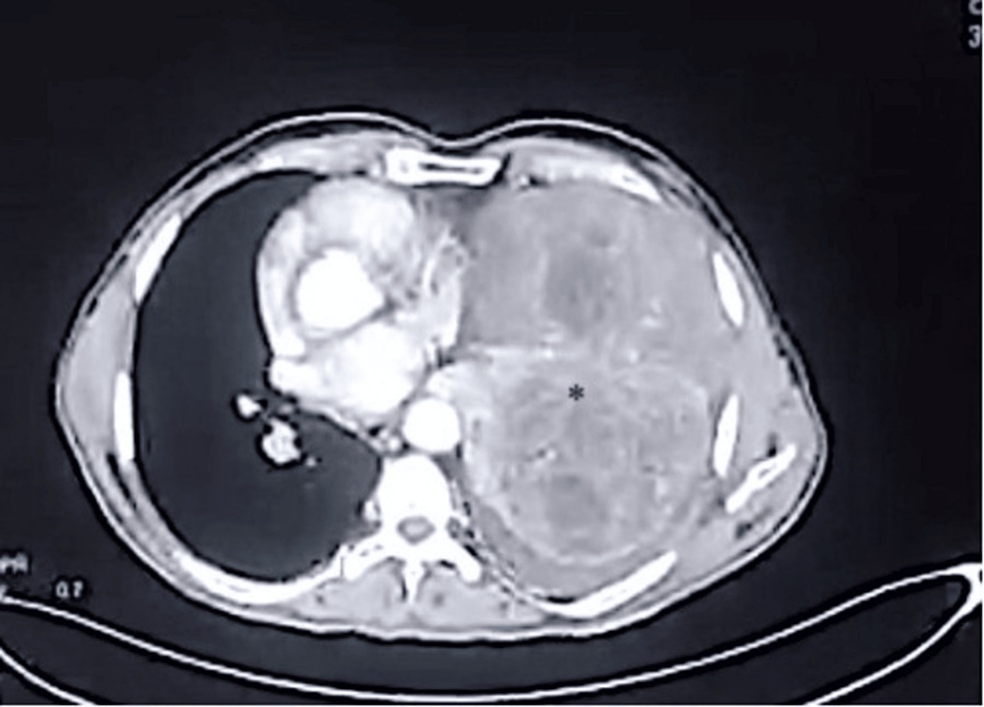
Contrast-enhanced computed tomography of the thorax shows a heterogeneously enhancing mass lesion in the left lung parenchyma, almost entirely replacing the left lung parenchyma (highlighted by asterisk).

A 76-year-old man presented with intermittent episodes of confusion and syncopal attacks (>10 episodes) for six months. The patient was a non-smoker, a non-alcoholic, and a farmer by profession. There was no history of fever, cough, shortness of breath, hemoptysis, chest pain, weight loss, or loss of appetite. There was no known history of diabetes mellitus, hypertension, or oral hypoglycemic medication. Auscultation revealed decreased left-sided air entry. His cardiovascular, abdominal, and central nervous system examination was unremarkable. At the time of presentation, his fasting blood sugar level was 38 mg/dl, his serum insulin level was <1 µU/mL (normal range: 4-16 µU/mL), and his C-peptide level was 0.11 ng/mL (normal range: 1.07-3.51 ng/mL). Serum IGF-2 level was not performed in this case. Chest X-ray revealed a left-sided homogenous opacity. Contrast-enhanced computed tomography (CECT) of the thorax showed a heterogeneously enhancing mass lesion in the left lung parenchyma measuring 16x15.2x15.9 cm in its maximum dimension, almost completely replacing the left lung parenchyma. There was no calcification or air foci within the lesion (Figure 1).

**Figure 2:**
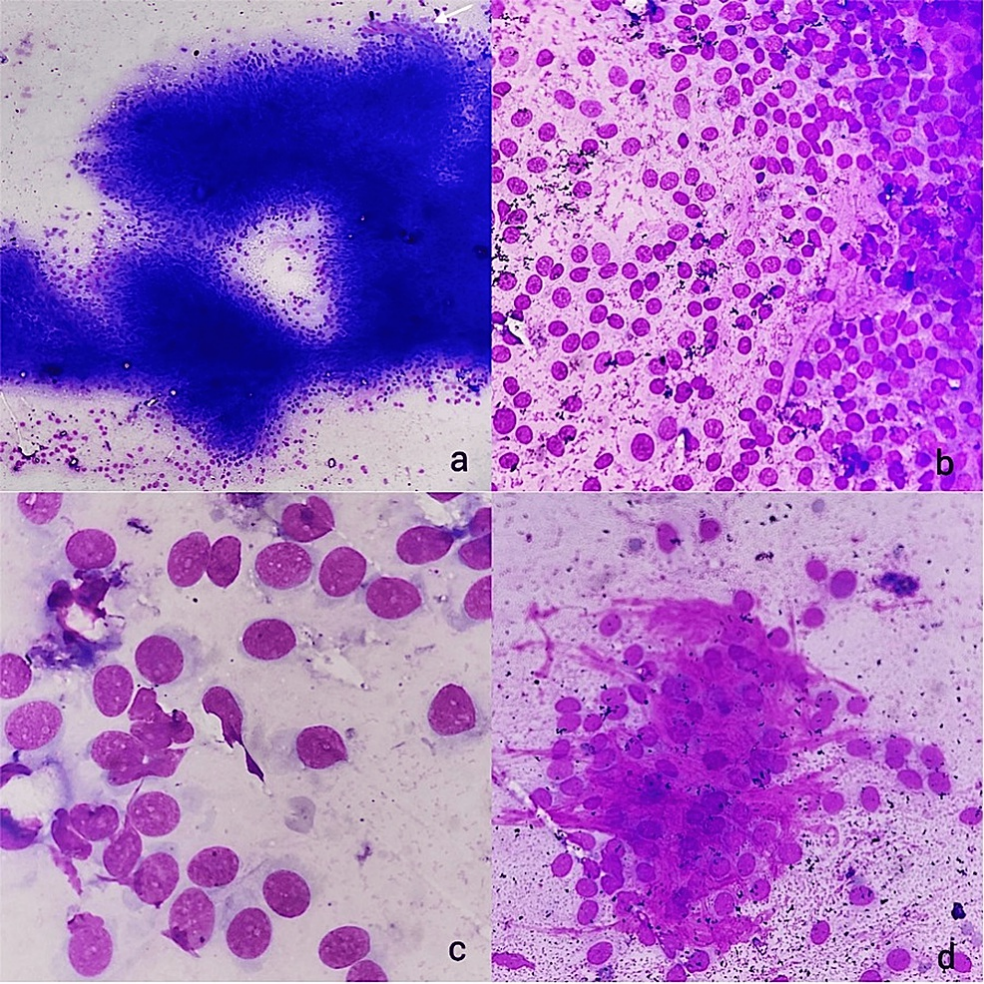
(a) Imprint smears showing fragments of tumor cells with traversing capillaries (white arrow) (10× May-Grünwald Giemsa stain). (b) Single cells and a few stripped nuclei smeared out from the fragments (20× May-Grünwald Giemsa stain). (c) Tumor cells showing monomorphic plump to oval nuclei, fine granular evenly dispersed chromatin, regular nuclear membranes, and inconspicuous nucleoli with a moderate amount of cytoplasm (40× May-Grünwald Giemsa stain). (d) Intercellular ropy collagenous matrix (20× May-Grünwald Giemsa stain).

A biopsy was performed, and imprint smears were submitted for cytology. The imprint smears were moderately cellular and showed tumor cells with monomorphic plump to oval nuclei arranged in clusters and fragments with traversing capillaries. Tumor cells had monomorphic plump to oval nuclei, fine granular evenly dispersed chromatin, regular nuclear membrane, and inconspicuous nucleoli with a moderate amount of focally wispy cytoplasm. Single cells and a few stripped nuclei were seen being smeared out from the fragments. No mitosis or necrosis was seen on the cytosmears. Intercellular collagenous matrix was seen in many of the fragments (Figure 2). A cytodiagnosis of low-grade mesenchymal neoplasm was made.

**Figure 3:**
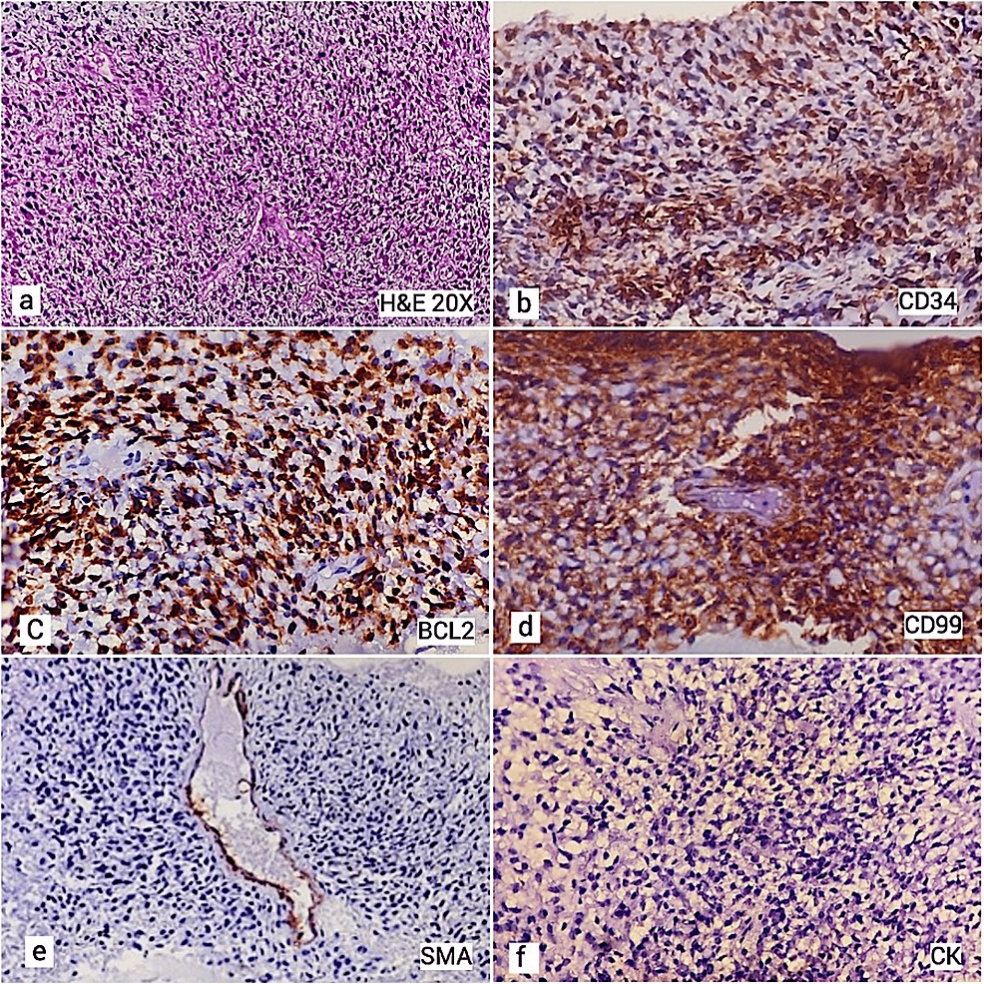
(a) Sections showing cellular tumor comprising of round to fusiform cells, arranged around blood vessels with intervening collagen (20× hematoxylin and eosin). (b) Immunohistochemistry showing positivity for CD34. (c) Immunohistochemistry showing positivity for BCL2. (d) Immunohistochemistry showing positivity for CD99. (e) Negative immunohistochemistry for SMA. (f) Negative immunohistochemistry for cytokeratin.

Biopsy showed a cellular tumor comprising round to fusiform cells, arranged around blood vessels with intervening thick ropy collagen. The cells displayed fusiform to spindled nuclei with mild pleomorphism. No mitosis or necrosis was seen. The tumor cells were positive for immunohistochemistry (IHC) for CD99, vimentin, BCL2, CD34, and STAT6 and negative for EMA, CK AE1/AE3, S100, TLE1, and SMA (Figure 3).

A diagnosis of SFT with NICTH (Doege-Potter syndrome) was made. During hospitalization, the patient's hypoglycemia was initially managed with 25 g of 50% intravenous dextrose. The patient continued to experience further episodes of spontaneous hypoglycemia requiring intravenous infusions of 10% dextrose. He was started on 60 mg of oral prednisone once daily. The patient was advised and counselled for surgery and further medical therapy, which the patient declined. He was discharged with an advice for regular glucose monitoring, caloric intake modification, and follow-up. The patient eventually succumbed to complications.

## Discussion

SFT is a rare fibroblastic neoplasm. Initially thought to be originating from the pleural mesothelium, it is now proved to be of mesenchymal origin. It usually arises from the visceral pleura but may occur within the lung parenchyma, pericardium, mediastinum, meninges, deep soft tissues, and other extrapulmonary locations. Pleuropulmonary SFT cases may remain clinically asymptomatic or may present with chest pain, cough, and shortness of breath.

Patients with SFT may occasionally present with paraneoplastic syndrome, most commonly hypoglycemic episodes, which may be the only presenting symptom of SFT, as was seen in the present case. Doege-Potter syndrome is characterized by refractory NICTH. It is seen in less than 5% of SFT cases, is particularly associated with large tumors, and is caused by the tumor secretion of IGF-2 [3,4]. In contrast to hyperinsulinemic hypoglycemia, patients with NICTH have low serum insulin and C-peptide concentrations during hypoglycemia. Other biochemical findings include low beta-hydroxybutyrate, elevated IGF-2/IGF-1 ratio (>3), and negative oral hypoglycemic agent screening [6]. NICTH can be seen associated with various other benign or malignant tumors as well [6]. Initial management of hypoglycemia is oral glucose and/or intravenous glucose or dextrose-containing fluids. The mainstay of therapy for NICTH is surgical resection of the underlying tumor. Complete removal of the tumor is curative for hypoglycemia in most cases. Increased caloric intake and medical management with glucocorticoids, glucagon, or recombinant human growth hormone (rhGH) are modes of treatment in cases where the tumor cannot be resected [6].

Radiographically, SFT appears as a well-circumscribed, usually homogenous soft tissue mass, with homogenous or heterogenous contrast enhancement. Large tumors may show cystic areas, calcifications, myxoid degeneration, or hemorrhage [7].

Cytomorphological features of SFT on fine needle aspiration cytology (FNAC) have been described in several studies. In this report, we present the cytomorphological features of SFT on imprint smears. In the present case, we found cells with round to oval bland nuclei, delicate capillaries within tissue fragments, and ropy collagen as important cytological features of SFT. Similar features have been highlighted in other case reports and series, including low to high cellularity, spindled to oval cells in clusters and fragments, with bland uniform chromatin, scant cytoplasm, and the presence of collagenous matrix [5,8-12]. Several authors have reported the presence of inconspicuous nucleoli in some cases, as was seen in the present case [5,8]. Traversing capillaries, as observed in the present case, have been described as an uncommon occurrence in most studies, though a few have reported similar findings. The presence of capillaries provides an important clue to diagnosis, replicating the histological appearance of hemangiopericytomatous vasculature [5,9,10]. 

Malignant features of SFT have been described in a few studies. Ali et al. described hypercellular smears, single cells with nuclear pleomorphism, and prominent nucleoli as important clues to malignancy [7]. Cho et al. reported that malignant SFTs showed a greater number of cells in clusters, and displayed mitotic activity, without significant cytological atypia [13]. Okada et al. described highly atypical epithelioid cells and mitotic figures in malignant cases of SFT [14]. In another study, Bishop et al. reviewed and studied cytological features in 13 cases of malignant SFT and reported hypercellularity, occasional prominent nucleoli, lack of single cells, focal pleomorphism, occasional mitosis, and rare necrosis as salient characteristics in their cases [15].

Differential diagnoses on cytology, particularly in the lung, may include small cell carcinoma, carcinoid, synovial sarcoma, sarcomatoid mesothelioma, myoepithelioma, inflammatory myofibroblastic tumor, fibromatosis, and malignant peripheral nerve sheath tumor (MPNST) [16-20]. In addition to differentiating cytomorphological features, preparation of cell blocks, immunocytochemistry (ICC), and immunohistochemistry (IHC) can aid in diagnosis (Table 1) [16-20].

**Table 1:** Table comparing the cytomorphological features and useful markers for the differential diagnosis of SFT. References: [16-20] N:C: nucleus-to-cytoplasmic; SFT: solitary fibrous tumor

Differential diagnosis	Cytomorphological differentiating features	Useful Immunocytochemistry/immunohistochemistry markers
Small cell carcinoma	Round to oval cells with high N:C ratio, hyperchromasia, finely dispersed chromatin, no distinct nucleoli, and minimal cytoplasm. Molding, crushing, high mitotic rate.	Synaptophysin, chromogranin, ki-67>30%
Carcinoid	Monomorphic small round/elongated or plasmacytoid tumor cells arranged in loose groups or singly dispersed, sometimes around branching capillaries, rosette-like structures. Smooth nuclear contour, with salt and pepper chromatin and a small nucleolus; no or rare mitoses. Absence of molding, necrosis, or nuclear crushing.	Synaptophysin, chromogranin, CK AE1/AE3
Monophasic synovial sarcoma	Cellular and shows a pericapillary arrangement of oval to round cells along with the presence of mast cells. The cells are highly pleomorphic compared to SFT.	EMA/cytokeratin, TLE1
SFT	Low to moderate cellularity. Oval to elongate, rounded, or stellate cells with wispy cytoplasm. Intercellular ropy collagenous matrix.	STAT 6 (nuclear positivity), CD34, Bcl2
Sarcomatoid mesothelioma	Loosely cohesive sheets of obviously malignant spindle cells. Poorly cellular smears of atypical spindle cells with a few fragments of collagen strands.	D2-20, calretinin, CK AE1/AE3
Inflammatory myofibroblastic tumor	Hypocellular smears with several small clusters of spindle cells with bland nuclei, small nucleoli, and occasional myxoid matrix. Prominent inflammatory component composed of lymphocytes and plasma cells.	ALK
Malignant peripheral nerve sheath tumor	Highly cellular, single as well as syncytial and three-dimensional clusters.	S100
Fibromatosis	Bland spindle cells with elongated nuclei embedded in metachromatic matrix material.	SMA, beta-catenin (nuclear expression), cyclin D1

## Conclusions

Doege-Potter syndrome is often incidentally diagnosed during the workup of hypoglycemia of uncertain etiology. Patients presenting with hypoglycemia without any prior history of diabetes mellitus should be evaluated for possible underlying neoplasm. Although the cytological diagnosis of an SFT is challenging, the presence of distinctive features like cells with round to oval bland nuclei, delicate capillaries within tissue fragments, and ropy collagen may provide clues to clinch the diagnosis in an appropriate clinical setting.
